# Quality improvement education for medical students: a near-peer pilot study

**DOI:** 10.1186/s12909-020-02020-9

**Published:** 2020-04-25

**Authors:** Elizabeth McGeorge, Charles Coughlan, Martha Fawcett, Robert Edward Klaber

**Affiliations:** Imperial College Healthcare NHS Trust, London, UK

**Keywords:** (3–10) – near-peer, Undergraduate, Quality improvement, Junior doctor

## Abstract

**Background:**

Quality improvement (QI) is an essential component of modern clinical practice. Front-line professionals offer valuable perspectives on areas for improvement and are motivated to deliver change. In the UK, all junior doctors are expected to participate in QI in order to advance to the next stage of their training. However, UK undergraduates receive no standardized training in QI methods. This is perpetuated within medical schools by a lack of teaching capacity and competing priorities, and may lead to tokenistic engagement with future QI projects.

**Methods:**

We describe a near-peer teaching programme designed to introduce students to QI methods. This pilot study was conceived and delivered in full by junior doctors and used existing resources to ensure high quality teaching content. 111 fifth-year medical students from the University of Cambridge were taught in interactive, participative workshops that encourage them to develop their own QI change ideas and projects. Core topics included the model for improvement, driver diagrams, stakeholder engagement, measurement for improvement and analysing and presenting data. Students completed surveys before and immediately after this intervention to assess their understanding of and confidence in utilizing QI methods. Questionnaires were also completed by junior doctor tutors.

**Results:**

Analysis of questionnaires completed before and immediately after the intervention revealed statistically significant improvements in students’ self-reported understanding of QI (*p* < 0.05) and confidence in applying techniques to their own work (p < 0.05). Students expressed a preference for QI teaching delivered by junior doctors, citing a relaxed learning environment and greater relevance to their stage of training. Tutors reported increased confidence in using QI techniques and a greater willingness to engage with QI in future.

**Conclusions:**

In this single-centre study, near-peer teaching produced significant improvements in students’ self-reported understanding of QI and confidence in applying QI methods. Near-peer teaching may constitute a sustainable means of teaching essential QI skills at undergraduate level. Future work must evaluate objective measures of student engagement with and competence in conducting QI.

## Background

Quality improvement (QI) aims to improve the safety, patient-centredness, efficacy, effectiveness, timeliness and equity of healthcare [1]. QI is increasingly viewed as a crucial part of medical education, equipping junior doctors with the skills to enhance patient experience and outcomes, improve population health, and reduce per capita cost of healthcare [2]. Medical students and junior doctors (practising doctors who have not yet completed specialist postgraduate training) have unique insights into the problems and opportunities within their organisations and how these might be met. Students have been recognised as a group with the time, space and motivation to conduct and champion quality improvement [3]. The UK General Medical Council, the regulator responsible for assuring the quality of medical school curricula in the UK, calls for all newly qualified doctors to be able to ‘apply the principles and methods of quality improvement.’ [4]. However, many junior doctors fail to complete mandatory QI projects during their training [5], citing poor knowledge of QI methods [6]. This is unsurprising given the lack of standardized and universal undergraduate QI teaching at UK medical schools [7].

There is no universal method to improve quality in healthcare and various overlapping approaches have been described [8]. The most widely used QI tool in healthcare settings is the ‘plan-do-study-act’ cycle [9]. Those seeking to improve a clinical pathway prospectively identify ‘change ideas’ and measures of interest (plan); implement an intervention (do); measure subsequent changes in outcomes of interest (study); and then scale up the intervention or switch to an alternative change idea based on the results (act). Iterative PDSA cycles provide a structured approach to translating ideas into action and enable rapid identification of effective interventions [10]. Other basic QI skills include the use of ‘action-effect’ (driver) diagrams to visualize actions needed to achieve project goals, and stakeholder mapping to identify individuals who will be affected by or influence the success of the project [8]. Examples of ongoing junior doctor-led QI projects at our National Health Service (NHS) Hospital Trust drawing on these skills include evaluations of:

- The impact of an intervention bundle on blood culture contamination rate
- Compliance with the World Health Organisation checklist in image-guided procedures
- Delivery of pain management for patients after major gynaecological surgery

QI teaching in universities is hampered by competing priorities within medical curricula and a lack of faculty teaching capacity [11, 12]. Theory-based teaching from senior QI experts can seem irrelevant and insignificant when compared to the major priority of students and educators – developing the clinical knowledge and skills needed to succeed as a doctor. Students may not understand why QI should be a priority or how they can have an impact as architects of change.

Near-peer teaching - delivered by tutors who are close to students in terms of training and experience - offers advantages over traditional senior led teaching. Taught material can seem more applicable and relevant to students [13] and rolling models for student development into tutors can create sustainable teaching programmes [14]. Tutors also benefit by developing teaching skills and consolidating their own knowledge [15]. Quality assurance is an important consideration in these programmes, and development of teaching materials and oversight by senior clinicians and educators is recommended [16].

The three junior doctor authors of this study all attended different UK medical schools and received little or no undergraduate teaching in QI methods. After participating in local QI training workshops for healthcare professionals, and becoming involved in various QI projects, we sought to cascade our learning to colleagues and medical students. We therefore developed a near-peer teaching pilot study to provide fifth-year medical students at the University of Cambridge with the skills and knowledge needed to conduct impactful QI projects. Our primary objective was to determine whether near-peer QI education could improve self-reported understanding of QI and confidence in applying QI techniques among medical students. Our secondary objective was to provide junior doctors with experience in near-peer education and teaching quality improvement techniques.

## Method

**Fig. 1:**
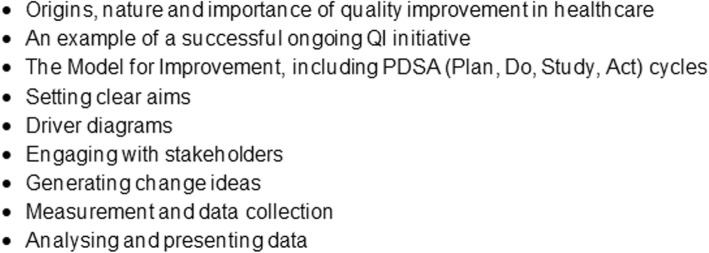
Topics covered during QI teaching workshop

A three-hour QI workshop was developed by three Academic Foundation Trainee (AFT) doctors in their second year of postgraduate training, after attending QI workshops hosted by staff at Imperial College Healthcare NHS Trust (ICHT). The workshop was based on ‘Tools for Change’ resources designed by the ICHT QI team. These included electronic slides and resources to support basic practical exercises, such as playing cards and flipcharts. Interactivity and early career relevance were prioritized, with didactic teaching interspersed with activities designed to develop students’ QI skills. All 276 fifth year students were invited to attend the workshops by senior faculty members at the medical school. Students were informed of the nature, purpose, format and our intent to evaluate these workshops by their course administrators. All students had previously received formal training in QI methods from senior members of University staff. Students were encouraged to bring project ideas to the workshops. Topics covered during the session are shown in Fig. 1. A full itemized summary of the workshop structure is shown in Supplementary Figure 1 (appendix).

Teaching was delivered by 8 junior doctors, each with 1–5 years of postgraduate experience. All tutors had direct experience of participation in audit and QI projects and had either attended QI teaching sessions at ICHT or received training from the three AFT doctors piloting the initiative. Tutors were recruited from amongst the personal and professional networks of the three doctors who designed the session and delivered the workshops in an unpaid voluntary capacity. Tutors were encouraged to draw on their own experiences to deliver practical advice alongside theoretical concepts. In January 2019, 49 students were taught in two simultaneous workshops run by three junior doctors. In March, a further 62 students were divided into six groups and taught by seven junior doctors.

We sought formal, structured feedback from students and tutors with a view to improving subsequent iterations.Students in both cohorts completed electronic pre- and post-workshop questionnaires. Questionnaires included an integrated summary explaining how students’ data would be used. Students were asked to reflect on the usefulness and content of the workshops. Questions relating to student confidence incorporated categorical Likert scales. Students were also invited to suggest improvements and enter free-text responses to provide a more rounded assessment of their experience. Tutors were asked to complete short, structured online surveys 6 weeks after the final workshop.

In accordance with guidelines from the University of Cambridge, we discussed the ethical implications of our project with the senior doctor responsible for the delivery of the leadership and management curriculum at the School of Clinical Medicine. This individual is also a member of the University’s Research Ethics Committee (REC). We were advised that this project constituted an educational evaluation, lay outside of the scope of the local REC and did not require formal ethical approval. Consent for the publication of anonymised, aggregated data from the evaluation was obtained from all students via the electronic questionnaires. Consent was withheld by one student whose data have been removed from the analysis.

## Results

Pre-session questionnaires were completed by 99/111 students (89%). Post-questionnaire responses were completed by 84/111 attendees (76%). Likert scales (rated 1 to 5 for ‘No confidence’ to ‘Very confident’) were analysed for significance using the Mann Whitney U test, with the null hypothesis of no increase in student confidence following the teaching session.

### Student understanding of QI and confidence in using QI techniques

**Fig. 2:**
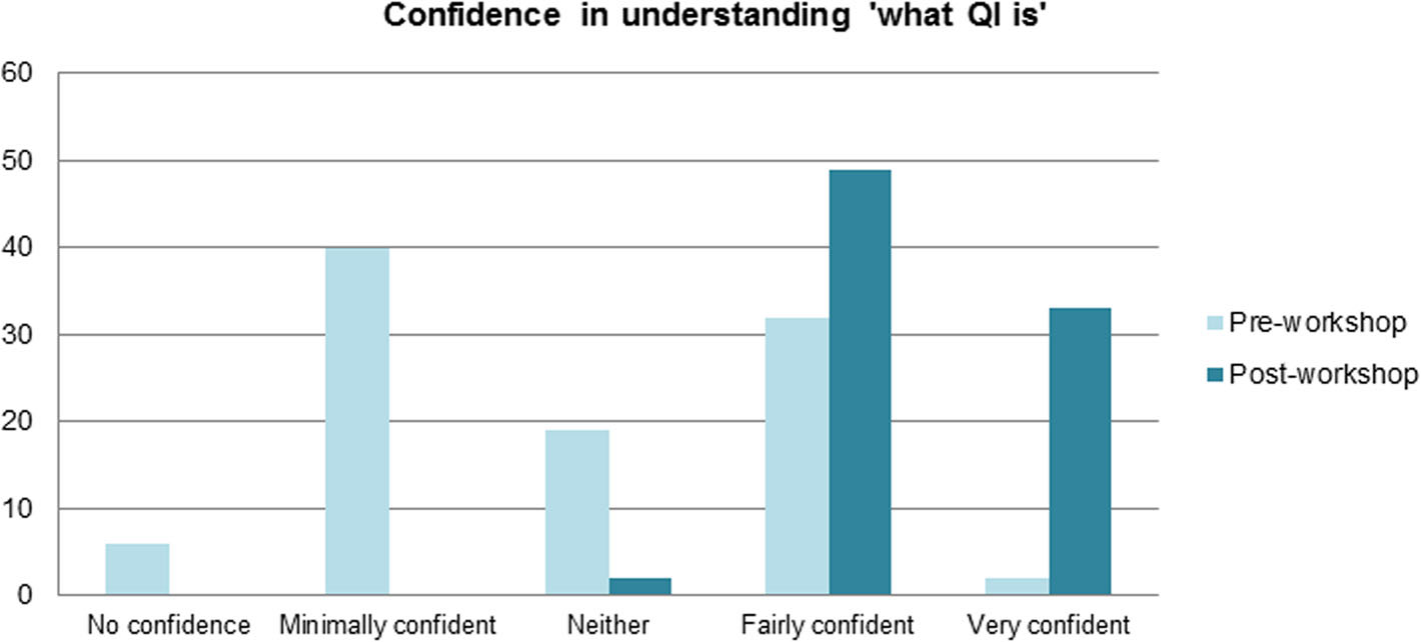
Student confidence in understanding of ‘what QI is’ before and after workshop

Confidence in understanding of QI techniques increased following teaching (*p* < 0.05), with mean Likert scores for the question ‘How confident are you in your understanding of what QI is?’ rising from 2.8 to 4.4 (Fig. 2). Following the session, 98% of students reported being ‘Fairly confident’ or ‘Very confident’ in their understanding of QI.

**Fig. 3:**
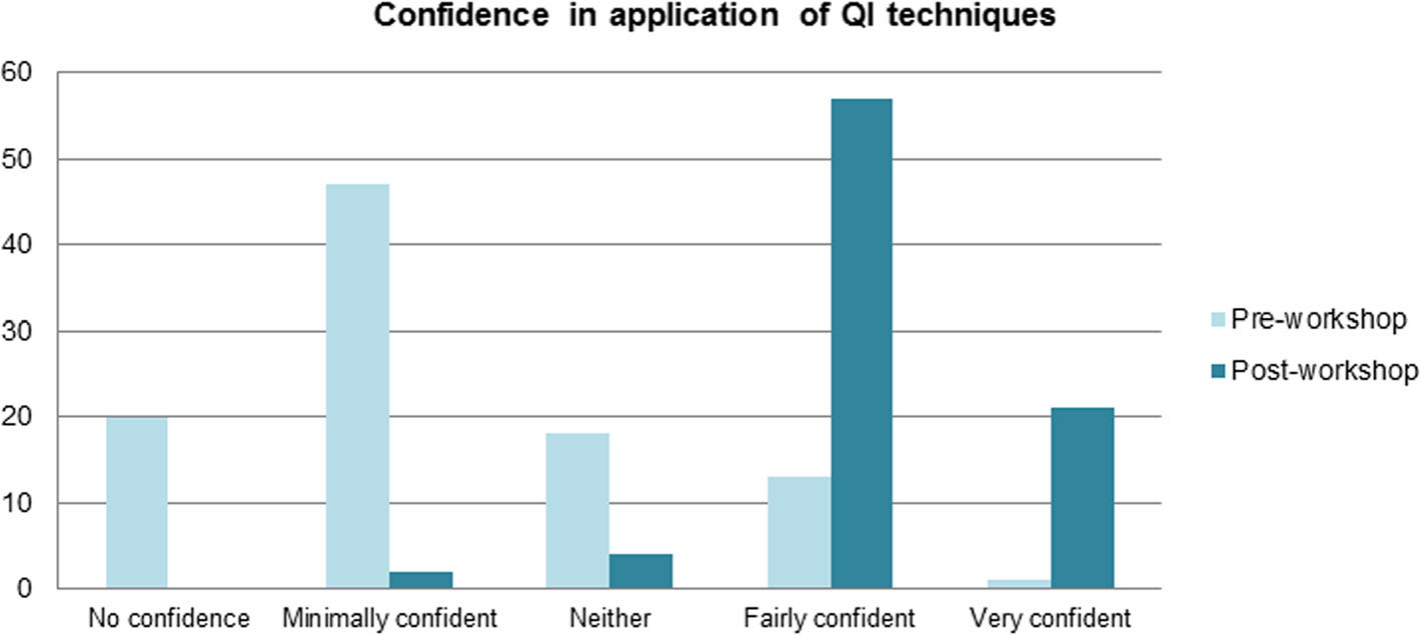
Student confidence in application of QI techniques before and after workshop

Confidence in applying QI techniques also increased following teaching (< 0.05), with mean Likert scores for the question ‘How confident are you in applying QI techniques to your own project?’ increasing from 2.3 to 4.1 (Fig. 3). 93% of students reported being ‘Fairly confident’ or ‘Very confident’ in response to this question.

### Student feedback

Only one third of students recalled any previous formal QI teaching. Students’ prior expectations focused on gaining an understanding of QI, including how to practically carry projects out and how to fit them into their careers. Following the teaching, 81% said that the workshop would be a useful addition to their curriculum.

Due to the quantity and quality of free-text responses, it was not possible to perform thematic analysis. We therefore present data from individual feedback forms to provide a snapshot of student opinion on the workshop. Students highlighted several positive factors, including the interactive teaching style, the licence for students to develop their own projects, and the mix of didactic teaching and group-based activities. Students suggested that the session could be shortened (though feedback was mixed with some requesting longer workshops) and positioned earlier within the medical school curriculum to enable early engagement with QI. Detail deemed to be superfluous by students attending the first session – such as the creation of Statistical Process Control charts – was removed before the second.

### Student perceptions of near-peer teaching

**Fig. 4:**
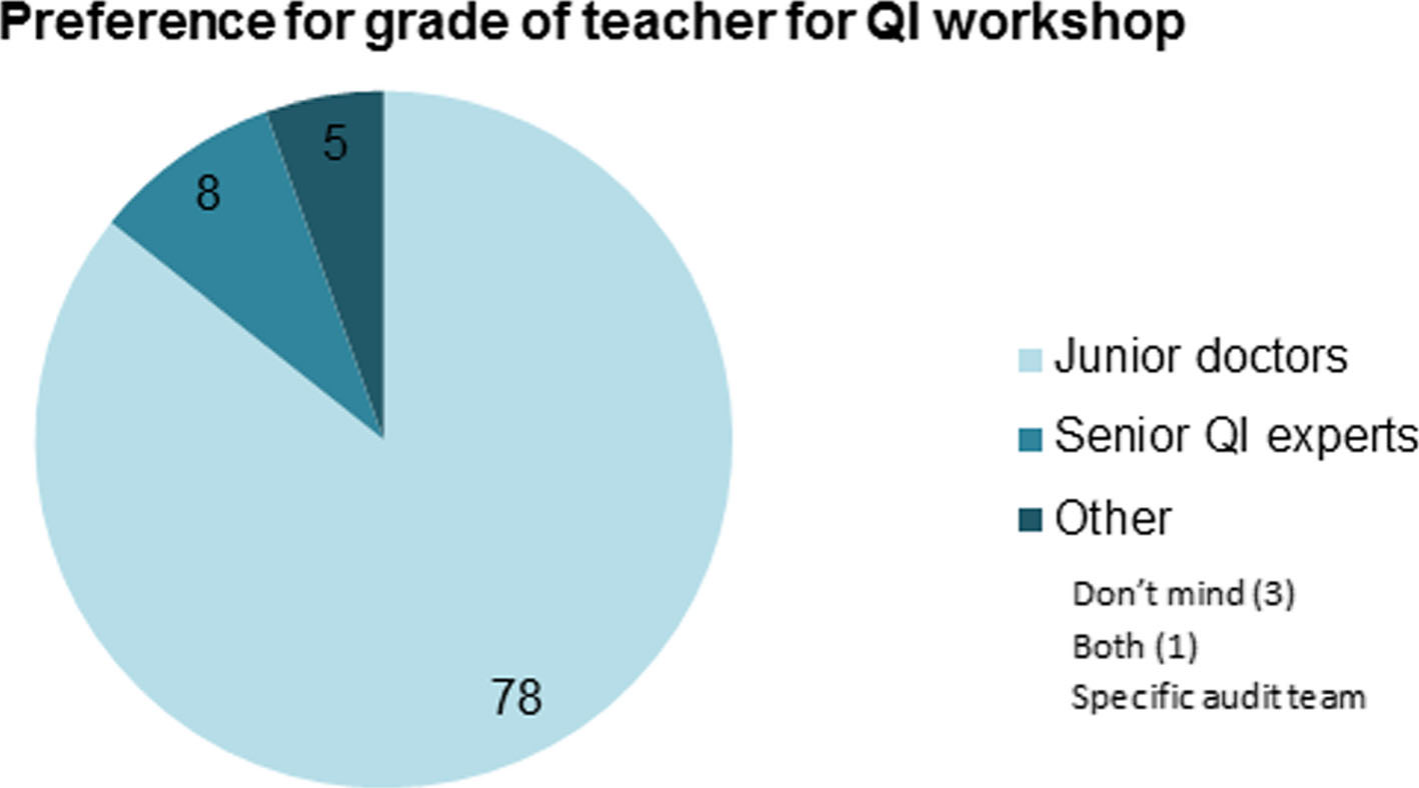
Student preferences for grade of teacher for QI sessions

Students were positive about being taught by junior doctors. 86% expressed a preference for being taught by junior doctors over ‘senior QI experts’ (Fig. 4). Detailed feedback from students revealed that this preference was underpinned by the proximity of junior doctors to undergraduate level and greater appreciation of the relevance of quality improvement to students. Other reasons cited by students included near-peer tutors being ‘more relatable’, approachable and ‘less daunting’ and tutors’ recent first-hand experiences of participation in QI within routine clinical practice. Some students commented that a mixture of junior doctors and ‘senior experts’ may be helpful when it came learning about and applying advanced QI techniques specific to their own projects.

### Tutor feedback

The junior doctors who delivered the sessions unanimously agreed that participation in this programme had improved their confidence in teaching and their knowledge of QI techniques; that following these sessions they would be more likely to engage in and initiate QI initiatives; and that it would be beneficial for junior doctors to regularly engage in QI teaching. Tutors – all with prior experience of teaching medical students in clinical and non-clinical settings – also reported high levels of student engagement with this project.

## Discussion

This pilot project was well received by students, tutors and educators at the University. Analysis of pre- and post-workshop student questionnaires demonstrated statistically significant improvements in students’ self-reported understanding of QI methods and confidence in applying them to their own work. Students valued the interactivity and direct application of QI methods to their own projects, with 86% expressing a preference for near-peer teaching, citing increased relevance to their stage of learning.

Junior doctors also benefited from involvement. The post-graduate curriculum encourages the acquisition of skills in QI, teaching and leadership alongside clinical competencies. Foundation doctors in particular are ‘expected to acquire and develop the skills needed to deliver teaching and mentoring effectively’ [17]. Our initiative promoted the development of teaching and QI capabilities amongst trainees. The steady supply of motivated junior doctors at teaching hospitals across the UK suggests that this approach could be made sustainable [18]. Standards can be maintained by using materials developed by senior staff [16]. Other forms of near-peer teaching have been shown to be non-inferior to teaching by faculty staff [19, 20]. This model can provide valuable teaching opportunities for junior doctors and ease pressures on overburdened faculty members.

### Limitations

We recognise that the recruitment of tutors from within our personal and professional network could introduce selection bias. Motivated volunteers may not represent the general junior doctor population [21]. We made a pragmatic decision to recruit doctors from within this network for the purposes of this pilot study; recruitment strategies will be reviewed for subsequent iterations of this work.

An extended evaluation with follow-up questionnaires 6–12 months after the workshops would have helped us to determine if improvements in self-reported outcomes were maintained over time. Of note, in our study, only 33% of respondents recalled any previous QI teaching, although the entire cohort had been taught about the importance and methods of QI by senior faculty earlier in their course. Temporal degradation in basic science knowledge [22] and complex skills [23] has been described in other groups of medical students. Unfortunately, extended evaluation was felt to be impractical as the three junior doctors responsible for the design, development and delivery of the workshops were working in busy clinical jobs over 60 miles from the study site.

We recognise that differences in group sizes and tutors’ experience of QI could lead to variation in tutor-student interactions and teaching content and quality. We took several steps to mitigate against this. The junior doctor co-authors of this work attended standardized teaching in QI for healthcare professionals from a dedicated QI team at their NHS organisation. This teaching was then cascaded down to the tutors who delivered the workshops. All workshops used a standardized format and materials and allowed sufficient time to support meaningful tutor-student interaction.

This study is limited by its single-centre setting; further work must be performed to validate near-peer QI teaching in other medical schools. Nonetheless, our approach has been endorsed by senior faculty members at the University of Cambridge, who have formally incorporated near-peer QI teaching into the undergraduate curriculum.

### Lessons and recommendations

This pilot study identifies near-peer QI teaching as a low-cost, high-impact model which could be applied and up-scaled across the UK and internationally. Future work should directly compare undergraduate QI teaching by junior doctors and senior teaching faculty. Extended evaluation of near-peer QI teaching programmes would also help to determine if self-reported improvements in knowledge of QI and QI methods are maintained over time. Assessment of objective markers of engagement with QI – such as completion of postgraduate QI projects relative to peers from other medical schools – would add further weight to this approach.

Students are a group with the time, space and motivation to engage with QI projects [3]. Medical schools have a duty to equip their graduates with knowledge of and skills in QI. They can encourage student engagement with QI by incorporating projects into clinical placements and incentivizing participation through assessment. In our view, near-peer QI teaching would be best placed at the mid-way point in medical school curricula.students with sufficient time to become involved in and complete QI projects before graduation.

## Conclusion

Engaging students in QI activities can be difficult, with competing educational priorities and a perception of difficulty in combining QI projects with clinical work. Given their involvement in compulsory QI projects, junior doctors are well placed to deliver relevant training, and will benefit by gaining teaching and leadership experience. We propose this well-received pilot of near-peer teaching as a model for undergraduate training in the UK.

## Supplementary information

**Additional file 1: Figure S1.** Near-peer QI teaching workshop structure. (PPTX 54 kb)

## Data Availability

With the exception of data pertaining to one student who withheld consent for publication, all data generated during this study are included in the published article.

## Abbreviations

AFT: Academic Foundation Trainee
ICHT: Imperial College Healthcare NHS Trust
PDSA: Plan-do-study-act
QI: Quality improvement
REC: Research Ethics Committee
UK: United Kingdom
