# Supplementary figures and images for: Quality improvement education for medical students: a near-peer pilot study
Source: BMC Med Educ. 2020 Apr 25;20:128. doi: 10.1186/s12909-020-02020-9 (PMC7183591; doi:10.1186/s12909-020-02020-9)

## Slide 1
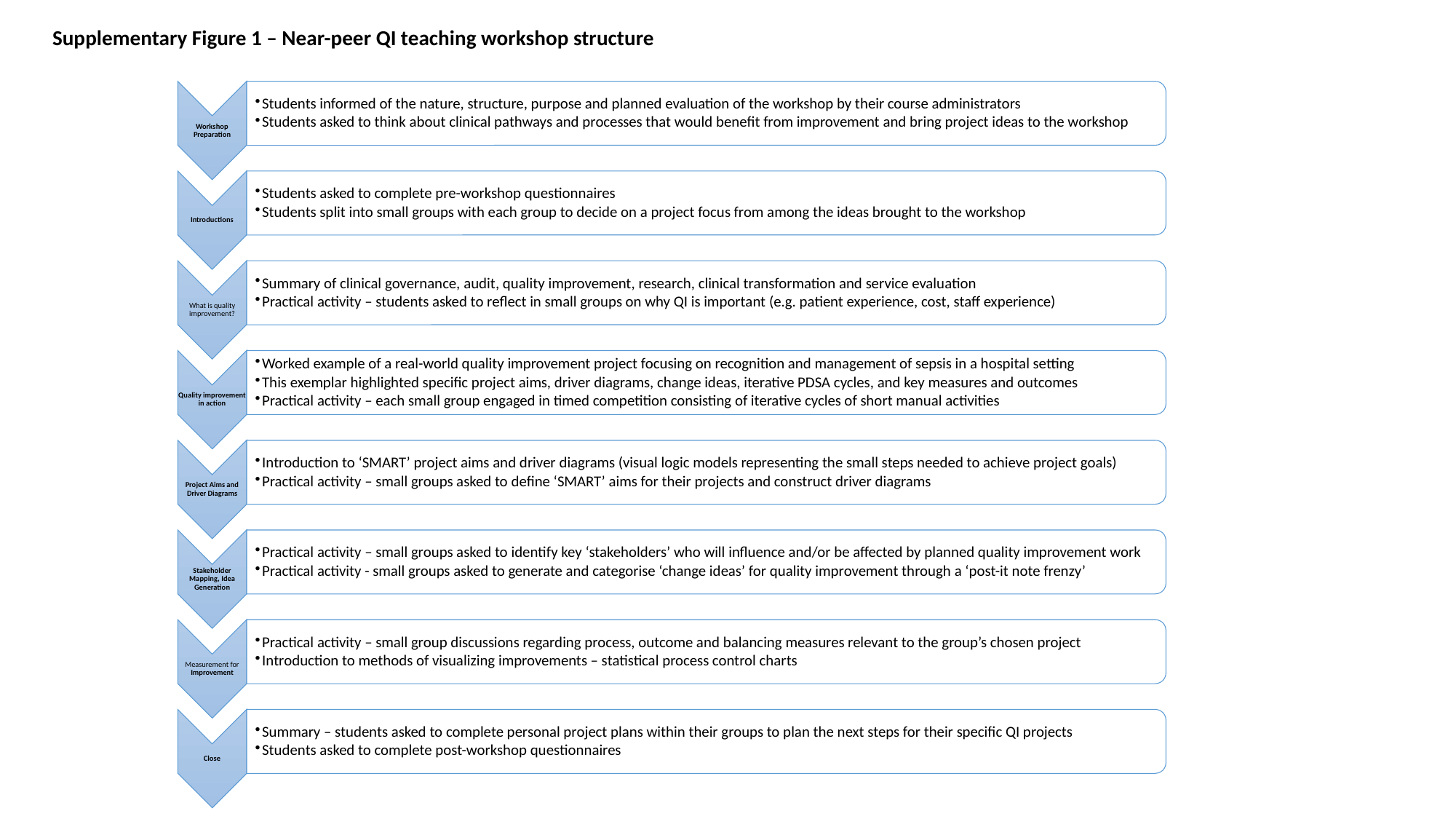

Supplementary Figure 1 – Near-peer QI teaching workshop structure

Supplement: Supplementary file 1 — Additional file 1: Figure S1. Near-peer QI teaching workshop structure. (PPTX 54 kb) [file 12909_2020_2020_MOESM1_ESM.pptx]
